# Self-reported acute respiratory illness during the pre-ritual and post-return phases of Hajj among South African pilgrims in 2025: a retrospective cohort study

**DOI:** 10.1016/j.ijregi.2026.101002

**Published:** 2026-09-03

**Authors:** Ozayr Mahomed, Salim Parker, Jaffar A. Al-Tawfiq

**Affiliations:** 1 Discipline of Public Health Medicine, School of Medicine, College of Health Sciences, University of KwaZulu-Natal, Durban, South Africa; 2 Private Practice and Division of Infectious Diseases, University of Cape Town, Cape Town, South Africa; 3 Specialty Internal Medicine and Quality Patient Safety Department, Johns Hopkins Aramco Healthcare, Dhahran, Saudi Arabia; 4 Infectious Diseases Division, Department of Medicine, Indiana University School of Medicine, Indianapolis, USA; 5 Infectious Diseases Division, Department of Medicine, Johns Hopkins University School of Medicine, Baltimore, USA

**Keywords:** Hajj, Acute respiratory illness, Mass gatherings, South African pilgrims, Influenza vaccination, Post-travel illness

## Abstract

**Objectives:**

Respiratory illness is common during Hajj, but phase-specific data from sub-Saharan African pilgrims remain limited. We examined self-reported acute respiratory illness (ARI) among South African pilgrims during the pre-ritual period in Saudi Arabia and within 14 days after return and assessed associated factors.

**Methods:**

This retrospective observational cohort study included South African pilgrims accredited for the 2025 (1446H) Hajj. A structured electronic questionnaire was administered approximately 1 month after Hajj to capture symptoms retrospectively over two predefined recall periods. The primary outcome was modified syndromic ARI, defined as two or more self-reported symptoms from cough, subjective fever, sore throat, rhinorrhea, or nasal congestion during the relevant recall period. Logistic regression estimated unadjusted odds ratios and adjusted odds ratios (aORs) with 95% confidence intervals (CIs).

**Results:**

Of approximately 2500 accredited pilgrims, 541 (21.6%) completed the questionnaire. During the pre-ritual period, 234/541 reported modified syndromic ARI (43.3%; 95% CI 39.1-47.5). Pilgrims aged ≥60 years had lower adjusted odds of pre-ritual ARI than those aged 18-39 years (aOR 0.47; 95% CI 0.26-0.87). Seasonal influenza vaccination was associated with higher odds of pre-ritual ARI (aOR 1.57; 95% CI 1.10-2.26). Within 14 days after return, 257/541 reported ARI (47.5%; 95% CI 43.2-51.8). Pre-ritual ARI remained modestly associated with post-return ARI in exploratory adjusted analysis (aOR 1.46; 95% CI 1.03-2.07), while influenza vaccination was not associated with post-return ARI.

**Conclusions:**

Modified syndromic ARI was common before the core Hajj rites and after return. Findings should not be interpreted as influenza-like illness, coronavirus disease 2019, pathogen-confirmed infection, or influenza vaccine effectiveness.

## Highlights

• Self-reported acute respiratory illness was common among South African Hajj pilgrims.
• Pre-ritual acute respiratory illness (ARI) affected 234/541 pilgrims (43.3%) before the core Hajj rites.
• Post-return ARI affected 257/541 pilgrims (47.5%) within 14 days of return.
• Pilgrims aged 60 years and older had lower adjusted odds of pre-ritual ARI.
• Influenza vaccination was not associated with post-return symptom-defined ARI.

## Introduction

The annual Hajj pilgrimage is one of the world’s largest recurring mass gatherings, bringing together pilgrims from diverse geographic regions in highly dense settings [1,2]. During Hajj, prolonged close contact, shared accommodation, crowding, physical exertion, and environmental stressors create conditions that facilitate the transmission of respiratory pathogens and place substantial demands on host-country health systems [2,3]. These risks have broader public health relevance because pilgrims return to countries across all regions of the world after the pilgrimage [1,3].

Respiratory tract infections are among the most frequently reported illnesses during and after Hajj [4,5]. Cohort studies and molecular surveillance have documented a high burden of acute respiratory illness (ARI) among pilgrims, with respiratory symptom attack rates often approaching or exceeding 50%, depending on case definition, timing of assessment, and diagnostic strategy [4,5]. Molecular studies further demonstrate the co-circulation of multiple respiratory pathogens, including influenza viruses, rhinoviruses, seasonal coronaviruses, respiratory syncytial virus, and bacterial pathogens such as *Streptococcus pneumoniae* and *Haemophilus influenzae* [5,6]. This pathogen diversity is important because syndromic respiratory illness during Hajj cannot be attributed to influenza alone and may reflect mixed viral and bacterial exposures.

Despite substantial global evidence, important population-level gaps remain. Most published Hajj respiratory studies have been conducted among pilgrims from Europe, Asia, the Middle East, and high-income settings, with comparatively limited data from sub-Saharan African pilgrims [7]. This is an important limitation because respiratory infection risk, comorbidity profiles, vaccination access, baseline exposure to respiratory pathogens, and health-seeking behavior may differ across regions. South African pilgrims represent a distinct cohort within this evidence gap, given the country’s background respiratory disease epidemiology, high burden of HIV-associated respiratory illness, and heterogeneous access to preventive health services [8]. Findings from other pilgrim populations may therefore not be directly generalizable to South African pilgrims.

A limited number of studies have examined morbidity among South African Hajj pilgrims. A survey of South African pilgrims attending the 2017 Hajj reported a substantial burden of respiratory illness during and after the pilgrimage [9]. A subsequent retrospective cohort study among South African pilgrims attending the 2023 Hajj similarly documented high post-Hajj morbidity, including respiratory symptoms after return [10]. However, few studies have examined symptom occurrence across clearly defined phases of the Hajj travel continuum, particularly the pre-ritual period in Saudi Arabia, defined as the period before commencement of the core 5-day Hajj rites, and the early post-return period.

The public health relevance of Hajj-associated respiratory illness extends beyond the pilgrimage itself. Returning pilgrims may introduce influenza and other respiratory viruses into their home communities, creating potential for onward transmission among household contacts and vulnerable groups [11]. Studies of returning Hajj and Umrah pilgrims have demonstrated respiratory viral infections after travel, supporting the value of post-travel surveillance and early clinical guidance [11]. However, data on illness before the core Hajj rites and on symptoms after return remain limited for African pilgrim cohorts.

Single-time-point assessments provide only a partial account of Hajj-associated respiratory illness. They may underestimate symptom duration, fail to distinguish illness occurring during different travel phases, and overlook symptoms that emerge after return. The present study addresses this gap by assessing self-reported ARI among South African pilgrims during the pre-ritual period in Saudi Arabia and within 14 days after return to South Africa. By examining phase-specific illness patterns and associated demographic, behavioral, clinical, and vaccination-related factors, this study provides population-specific evidence to inform pre-Hajj counseling, risk communication, and post-return public health guidance.Methods

### Study design and setting

We conducted a retrospective observational cohort study using post-Hajj self-reported symptom recall among South African pilgrims who participated in the 2025 (1446H) Hajj pilgrimage. Data were collected using a structured electronic questionnaire administered approximately 1 month after completion of Hajj.

The questionnaire captured symptoms retrospectively for two predefined recall periods: (1) the pre-ritual period in Saudi Arabia, defined as the period before commencement of the core 5-day Hajj rites, and (2) the first 14 days after return to South Africa. Because symptom data were collected after Hajj, the study estimates self-reported symptom occurrence during defined recall periods rather than prospectively observed incident infection.

The primary objective was to estimate the self-reported cumulative occurrence of modified syndromic ARI during these two periods. The secondary objective was to examine demographic, clinical, behavioral, functional, and vaccination-related factors associated with modified syndromic ARI in each period.

### Study population and eligibility

The study population comprised South African pilgrims accredited by the South African Hajj and Umrah Council (SAHUC) for the 1446H/2025 Hajj. Eligible participants were adults aged 18 years or older who had confirmed SAHUC accreditation and access to a smartphone or electronic device capable of completing the online questionnaire.

Participation was voluntary. All participants reviewed the study information electronically and provided digital informed consent before completing the questionnaire.

### Sampling strategy and sample size

South Africa’s accredited Hajj quota for 2025 was approximately 2500 pilgrims. The minimum required sample size was estimated at 259 participants, assuming an expected ARI prevalence of 25%, a 5% margin of error, a 95% confidence level, and finite population correction for the accredited pilgrim population.

To maximize participation, all accredited South African pilgrims were invited. Recruitment, therefore, followed a voluntary census-style approach rather than probabilistic sampling. The final analytical sample comprised 541 participants, representing 21.6% of the eligible accredited population.

Individual-level demographic, clinical, or vaccination data were not available for non-respondents; therefore, respondents could not be formally compared with the full accredited cohort. The final sample should therefore be interpreted as a voluntary respondent cohort rather than a probability sample. Because participation required access to an electronic device, digitally excluded pilgrims may have been underrepresented.

### Data collection instrument and variables

A structured, secure electronic questionnaire was developed and designed to capture variables commonly reported in Hajj respiratory illness studies and was reviewed by investigators with experience in Hajj health research before administration.

The questionnaire captured sociodemographic characteristics, clinical history, behavioral factors, functional indicators, vaccination history, and respiratory symptoms. Sociodemographic variables included age, sex, marital status, and education level. Clinical and behavioral variables included smoking history, self-reported weight status, pre-existing chronic medical condition status, and baseline self-rated health. Functional indicators included walking distance capacity, self-rated physical fitness, and physical training before Hajj. Chronic medical condition status was recorded as a self-reported binary variable indicating whether the participant had any pre-existing chronic medical condition; individual diagnoses were not captured separately. Obesity was not included in this composite variable and was analyzed separately as weight status.

Vaccination history focused on self-reported receipt of seasonal influenza vaccination before Hajj. South Africa uses the Southern Hemisphere seasonal influenza vaccine formulation, with vaccination generally offered before the winter influenza season, typically around March or April. In 2025, South Africa’s influenza season began in epidemiological week 13, starting 24 March 2025, overlapping with the pre-Hajj preparation period for many pilgrims [12,13]. Coronavirus disease 2019 (COVID-19) vaccination status was collected but excluded from analysis because of substantial missingness and inconsistent reporting that precluded reliable classification.

Participants reported whether they experienced cough, fever, sore throat, rhinorrhea, or nasal congestion during the pre-ritual period in Saudi Arabia and within 14 days after return to South Africa. Fever was self-reported and was not verified by temperature measurement. Closed-ended symptom options were used to reduce variability in symptom reporting.

No laboratory confirmation was performed. The ARI outcome, therefore, represents modified symptom-defined respiratory illness and should not be interpreted as pathogen-confirmed respiratory infection. The study was not designed to estimate influenza vaccine effectiveness.

### Case definition and outcomes

The primary outcome was modified syndromic ARI. ARI was defined as the presence of two or more self-reported symptoms during the relevant recall period from the following symptom list: cough, subjective fever, sore throat, rhinorrhea, or nasal congestion. Fever was based on participant self-report and was not objectively measured using a recorded temperature.

This definition was selected to improve specificity compared with a single-symptom definition while remaining feasible for retrospective self-report.

The outcome should not be interpreted as laboratory-confirmed respiratory infection. It was also not classified as influenza-like illness (ILI), because standard ILI surveillance definitions require acute respiratory infection with measured fever of ≥38°C and cough, with symptom onset within the last 10 days; these data were not collected in the present retrospective questionnaire [14].

The outcome was not classified as COVID-19 because laboratory testing, severe acute respiratory syndrome coronavirus 2 confirmation, and COVID-19 case-classification data were not available [15].

Two outcomes were analyzed separately:

1. self-reported modified syndromic ARI during the pre-ritual period in Saudi Arabia; and
2. self-reported modified syndromic ARI within 14 days after return to South Africa.

Self-reported cumulative occurrence proportions were calculated for each outcome period with 95% confidence intervals (CIs).

The post-return outcome was defined as self-reported modified syndromic ARI occurring within 14 days after return to South Africa. To examine symptom persistence or phase overlap, pre-ritual ARI was also evaluated as an explanatory variable for post-return ARI.

### Data management and statistical analysis

Survey data were exported to Microsoft Excel for validation and cleaning. Duplicate entries were removed, logical consistency checks were performed, and records with missing outcome data were excluded from the relevant analysis. Personal identifiers collected for quality control and duplicate checks were removed before analysis. All statistical analyses were conducted using de-identified data.

Participant characteristics were summarized using frequencies and percentages. Associations between explanatory variables and ARI were first examined using chi-square tests or Fisher exact tests where appropriate. Logistic regression was used to estimate unadjusted odds ratios (ORs) and adjusted odds ratios (aORs) with 95% CIs.

Multivariable models included variables selected *a priori* based on biological plausibility and prior Hajj respiratory illness literature, including age, sex, marital status, education level, smoking history, weight status, baseline self-rated health, chronic medical conditions, walking distance capacity, and influenza vaccination status. Variables with *P* < 0.20 in bivariate analyses were reviewed but were not used as the sole criterion for model inclusion.

Self-rated physical fitness and physical training before Hajj were examined descriptively and in bivariate analyses but were not retained in adjusted models because of collinearity or conceptual overlap with walking distance capacity. Walking distance capacity was retained as the functional-capacity measure. Collinearity among candidate variables was assessed using chi-square tests and Cramér *V*.

Complete-case analysis was applied, and the number of observations included in each analysis is reported in the relevant tables. For the post-return outcome, self-reported ARI during the pre-ritual period was also examined as an explanatory variable to assess whether earlier symptom reporting was associated with symptoms after return. The post-return multivariable model was treated as exploratory because of sparse subgroup data, wide CIs, and potential instability. Adjusted post-return estimates were therefore interpreted cautiously.

All analyses were conducted using Stata/Standard Edition version 18.5 (StataCorp, College Station, TX, USA). A two-sided *P*-value of <0.05 was considered statistically significant.

### Ethical considerations

Ethical approval was obtained from the Human and Social Sciences Research Ethics Committee of the University of KwaZulu-Natal (HSSREC/00008459/2025). Permission to access the accredited pilgrim cohort was granted by SAHUC.

Digital informed consent was obtained from all participants before completion of the questionnaire. Personal identifiers were used only for data linkage, duplicate checks, and quality control during data collection. Identifiers were stored separately from analytical data and removed after duplicate checks and quality-control procedures were completed. The final analytical dataset was fully anonymized before analysis to ensure participant confidentiality.Results

### Respiratory illness during the pre-ritual Hajj period in Saudi Arabia

#### Self-reported cumulative occurrence and participant characteristics

A total of 541 of 2500 accredited South African pilgrims participated in the study, giving a response proportion of 21.6%. During the pre-ritual period in Saudi Arabia, defined as the period before commencement of the core 5-day Hajj rites, 234/541 participants reported two or more self-reported symptoms meeting the modified syndromic ARI case definition (43.3%; 95% CI 39.1-47.5). The distribution of participant characteristics by pre-ritual ARI status is shown in Table 1.

**Table 1:** Participant characteristics by self-reported acute respiratory illness during the pre-ritual Hajj period in Saudi Arabia, South African pilgrims, 2025 (*n* = 541).

Characteristic	Total *n* (%)	ARI *n*/*N* (%)	No ARI *n*/*N* (%)	*P*-value
**Total**	541 (100)	234/541 (43.3)	307/541 (56.7)	—
**Sex**				0.771
Female	362 (66.9)	155/362 (42.8)	207/362 (57.2)	
Male	179 (33.1)	79/179 (44.1)	100/179 (55.9)	
**Age group (years)**				0.024
18-39	149 (27.5)	69/149 (46.3)	80/149 (53.7)	
40-49	136 (25.1)	69/136 (50.7)	67/136 (49.3)	
50-59	144 (26.6)	60/144 (41.7)	84/144 (58.3)	
≥60	112 (20.7)	36/112 (32.1)	76/112 (67.9)	
**Marital status**				0.496
Single	34 (6.3)	18/34 (52.9)	16/34 (47.1)	
Married	454 (83.9)	193/454 (42.5)	261/454 (57.5)	
Widowed/divorced	53 (9.8)	23/53 (43.4)	30/53 (56.6)	
**Education level**				0.141
School (primary/secondary)	190 (35.1)	75/190 (39.5)	115/190 (60.5)	
College/university	328 (60.6)	152/328 (46.3)	176/328 (53.7)	
Trade	23 (4.3)	7/23 (30.4)	16/23 (69.6)	
**Smoking history**				0.888
Never	302 (55.8)	129/302 (42.7)	173/302 (57.3)	
Current smoker	99 (18.3)	42/99 (42.4)	57/99 (57.6)	
Former smoker	140 (25.9)	63/140 (45.0)	77/140 (55.0)	
**Weight status**				0.142
Normal/underweight	341 (63.0)	140/341 (41.1)	201/341 (58.9)	
Overweight	186 (34.4)	90/186 (48.4)	96/186 (51.6)	
Obese	14 (2.6)	4/14 (28.6)	10/14 (71.4)	
**Baseline health rating**				0.573
Fair/poor	103 (19.0)	42/103 (40.8)	61/103 (59.2)	
Good/excellent	438 (81.0)	192/438 (43.8)	246/438 (56.2)	
**Chronic medical condition**				0.759
No	241 (44.6)	106/241 (44.0)	135/241 (56.0)	
Yes	300 (55.4)	128/300 (42.7)	172/300 (57.3)	
**Physical fitness**				0.674
Poor	181 (33.5)	76/181 (42.0)	105/181 (58.0)	
Good-exceptional	360 (66.5)	158/360 (43.9)	202/360 (56.1)	
**Walking distance capacity**				0.618
<2.5 km	80 (14.8)	35/80 (43.8)	45/80 (56.2)	
2.5-<5 km	130 (24.0)	62/130 (47.7)	68/130 (52.3)	
5-<10 km	178 (32.9)	76/178 (42.7)	102/178 (57.3)	
≥10 km	153 (28.3)	61/153 (39.9)	92/153 (60.1)	
**Physical training before Hajj**				0.072
No	175 (32.4)	66/175 (37.7)	109/175 (62.3)	
Yes	366 (67.6)	168/366 (45.9)	198/366 (54.1)	
**Seasonal influenza vaccination**				0.029
No	251 (46.4)	96/251 (38.2)	155/251 (61.8)	
Yes	290 (53.6)	138/290 (47.6)	152/290 (52.4)	

Values are presented as *n*/*N* (%) unless otherwise indicated. Percentages are row percentages. *P*-values were calculated using chi-square tests or Fisher exact tests where appropriate.

Abbreviation: ARI, acute respiratory illness.

Symptom occurrence did not differ significantly by sex, with symptoms reported by 155/362 (42.8%) female pilgrims and 79/179 (44.1%) male pilgrims (*P* = 0.771). Symptom occurrence differed significantly by age group (*P* = 0.024), with the highest proportion observed among pilgrims aged 40-49 years (69/136; 50.7%) and the lowest among those aged ≥60 years (36/112; 32.1%) (Table 1).

No statistically significant differences in symptom occurrence were observed by marital status, education level, smoking history, weight status, baseline health rating, chronic medical condition status, physical fitness, or walking distance capacity.

Physical training before Hajj showed a borderline, non-significant association with symptom occurrence. Symptoms were reported by 168/366 pilgrims who had engaged in physical training before Hajj compared with 66/175 who had not (45.9% vs 37.7%; *P* = 0.072). Seasonal influenza vaccination was significantly associated with symptom reporting: 138/290 vaccinated pilgrims reported symptoms compared with 96/251 unvaccinated pilgrims (47.6% vs 38.2%; *P* = 0.029).

### Factors associated with self-reported ARI during the pre-ritual Hajj period

#### Bivariate analysis

Unadjusted associations between participant characteristics and self-reported ARI are shown in Table 2. Age group was significantly associated with ARI reporting (χ²(3) = 9.45, *P* = 0.024). Compared with pilgrims aged 18-39 years, those aged ≥60 years had lower unadjusted odds of reporting ARI (OR 0.55; 95% CI 0.33-0.92). No statistically significant differences were observed for pilgrims aged 40-49 years or 50-59 years compared with the reference group.

**Table 2:** Unadjusted and adjusted associations between participant characteristics and self-reported acute respiratory illness during the pre-ritual Hajj period in Saudi Arabia, South African pilgrims, 2025.

Variable	Symptoms present *n*/*N* (%)	χ² (df)	*P*-value	Unadjusted OR (95% CI)	Adjusted OR^a^ (95% CI)
**Sex (female)**		0.09 (1)	0.771		
Male	79/179 (44.1)			1.06 (0.72-1.54)	1.22 (0.81-1.85)
**Age group (18-39 years)**		9.45 (3)	0.024		
40-49	69/136 (50.7)			1.19 (0.75-1.90)	1.14 (0.70-1.87)
50-59	60/144 (41.7)			0.83 (0.52-1.31)	0.76 (0.46-1.27)
≥60	36/112 (32.1)			0.55 (0.33-0.92)	0.47 (0.26-0.87)
**Marital status (single)**		1.40 (2)	0.496		
Married	193/454 (42.5)			0.66 (0.33-1.32)	0.66 (0.32-1.38)
Widowed/divorced	23/53 (43.4)			0.68 (0.29-1.62)	0.90 (0.35-2.30)
**Education level (school)**		3.92 (2)	0.141		
College/university	152/328 (46.3)			1.32 (0.92-1.90)	1.10 (0.72-1.68)
Trade	7/23 (30.4)			0.67 (0.26-1.71)	0.59 (0.22-1.56)
**Smoking history (never)**		0.24 (2)	0.888		
Current smoker	42/99 (42.4)			0.99 (0.62-1.56)	1.10 (0.66-1.83)
Former smoker	63/140 (45.0)			1.10 (0.73-1.64)	1.11 (0.72-1.72)
**Weight status (normal/underweight)**		3.90 (2)	0.142		
Overweight	90/186 (48.4)			1.35 (0.94-1.93)	1.29 (0.87-1.92)
Obese	4/14 (28.6)			0.57 (0.18-1.87)	0.52 (0.15-1.76)
**Baseline health rating (fair/poor)**		0.32 (1)	0.573		
Good/excellent	192/438 (43.8)			1.13 (0.72-1.80)	1.20 (0.75-1.92)
**Chronic medical condition (no)**		0.09 (1)	0.759		
Yes	128/300 (42.7)			0.95 (0.66-1.35)	1.00 (0.68-1.47)
**Walking distance capacity (<2.5 km)**		1.79 (3)	0.618		
2.5-<5 km	62/130 (47.7)			1.17 (0.67-2.05)	1.09 (0.60-1.99)
5-<10 km	76/178 (42.7)			0.96 (0.56-1.63)	0.85 (0.48-1.51)
≥10 km	61/153 (39.9)			0.85 (0.49-1.47)	0.66 (0.35-1.24)
**Physical training before Hajj (no)**		3.23 (1)	0.072		
Yes	168/366 (45.9)			1.40 (0.95-2.06)	—b
**Seasonal influenza vaccination (no)**		4.78 (1)	0.029		
Yes	138/290 (47.6)			1.47 (1.02-2.10)	1.57 (1.10-2.26)

Values are presented as *n*/*N* (%) unless otherwise indicated. Percentages are row percentages.

a Adjusted for sex, age group, marital status, education level, smoking history, weight status, baseline health rating, chronic medical condition status, walking distance capacity, and seasonal influenza vaccination.

b Physical training before Hajj was examined in bivariate analysis but was not retained in the adjusted model because of conceptual overlap with functional-capacity indicators. Self-reported physical fitness was excluded from multivariable modeling because of collinearity with walking distance capacity. Abbreviations: CI, confidence interval; df, degrees of freedom; OR, odds ratio.

Seasonal influenza vaccination was also associated with ARI reporting (χ²(1) = 4.78, *P* = 0.029). Vaccinated pilgrims had higher unadjusted odds of reporting symptoms than unvaccinated pilgrims (OR 1.47; 95% CI 1.02-2.10). Physical training before Hajj showed a borderline non-significant association with ARI reporting (χ²(1) = 3.23, *P* = 0.072), while walking distance capacity was not associated with ARI (χ²(3) = 1.79, *P* = 0.618).

No statistically significant associations were observed for sex, marital status, education level, smoking history, weight status, baseline self-rated health, or chronic medical condition status.

#### Multivariable analysis

In the adjusted logistic regression model, age group and seasonal influenza vaccination remained associated with self-reported ARI during the pre-ritual period in Saudi Arabia.

Pilgrims aged ≥60 years had lower adjusted odds of reporting ARI than those aged 18-39 years (aOR 0.47; 95% CI 0.26-0.87). No significant associations were found for sex, marital status, education level, smoking history, weight status, baseline health rating, chronic medical conditions, or walking capacity.

Seasonal influenza vaccination remained associated with higher adjusted odds of symptom-defined ARI (aOR 1.57; 95% CI 1.10-2.26). Because the outcome was syndromic and not laboratory-confirmed influenza, this association should be interpreted cautiously and should not be taken as evidence of influenza vaccine failure or harm.

Self-reported physical fitness was included in descriptive and bivariate analyses but excluded from multivariable modeling because of collinearity with walking distance capacity (χ²(3) = 77.9, *P* < 0.001; Cramér *V* = 0.38). Walking distance capacity was retained as the functional-capacity measure. Physical training before Hajj was examined in bivariate analysis but was not retained in the adjusted model because of conceptual overlap with functional-capacity indicators.

### Self-reported ARI within 14 days after return to South Africa

#### Self-reported cumulative occurrence and participant characteristics

Respiratory symptoms remained common after return to South Africa. Within 14 days after return, 257/541 pilgrims reported two or more self-reported symptoms meeting the modified syndromic ARI case definition, giving a self-reported cumulative occurrence of 47.5% (95% CI 43.2-51.8; Table 3).

**Table 3:** Unadjusted and exploratory adjusted associations between participant characteristics and self-reported acute respiratory illness within 14 days after return to South Africa, South African Hajj pilgrims, 2025.

Variable	Symptoms present *n*/*N* (%)	χ² (df)	*P*-value	Unadjusted OR (95% CI)	Adjusted OR^a^**(95% CI)**
**Sex (female)**		2.12 (1)	0.145		
Male	93/179 (52.0)			1.31 (0.90-1.90)	1.28 (0.89-1.84)
**Age group (18-39 years)**		2.29 (3)	0.515		
40-49	59/136 (43.4)			0.89 (0.56-1.42)	0.86 (0.54-1.39)
50-59	70/144 (48.6)			1.10 (0.69-1.74)	1.11 (0.69-1.80)
≥60	59/112 (52.7)			1.29 (0.79-2.11)	1.31 (0.77-2.23)
**Marital status (single)**		2.29 (2)	0.318		
Married	217/454 (47.8)			0.72 (0.36-1.46)	—
Widowed/divorced	21/53 (39.6)			0.52 (0.22-1.24)	—
**Education level (school)**		3.26 (2)	0.196		
College/university	162/328 (49.4)			1.13 (0.79-1.62)	—
Trade	7/23 (30.4)			0.51 (0.20-1.29)	—
**Smoking history (never)**		0.62 (2)	0.733		
Current smoker	45/99 (45.5)			0.87 (0.55-1.37)	—
Former smoker	64/140 (45.7)			0.88 (0.59-1.31)	—
**Weight status (normal/underweight)**		5.89 (2)	0.053		
Overweight	96/186 (51.6)			1.34 (0.94-1.92)	—
Obese	10/14 (71.4)			3.15 (0.97-10.23)	—
**Baseline health rating (fair/poor)**		0.80 (1)	0.372		
Good/excellent	204/438 (46.6)			0.82 (0.54-1.26)	—
**Chronic medical condition (no)**		0.01 (1)	0.929		
Yes	142/300 (47.3)			0.98 (0.69-1.40)	0.93 (0.65-1.34)
**Walking distance capacity (<2.5 km)**		5.98 (3)	0.113		
2.5-<5 km	73/130 (56.2)			1.49 (0.85-2.60)	—
5-<10 km	83/178 (46.6)			1.02 (0.60-1.72)	—
≥10 km	64/153 (41.8)			0.84 (0.48-1.44)	—
**Pre-ritual ARI (no)**		4.23 (1)	0.040		
Yes	123/234 (52.6)			1.43 (1.02-2.01)	1.46 (1.03-2.07)
**Seasonal influenza vaccination (no)**		2.02 (1)	0.155		
Yes	146/290 (50.3)			1.28 (0.90-1.82)	1.20 (0.85-1.71)

Values are presented as *n*/*N* (%) unless otherwise indicated. Percentages are row percentages. *P*-values were calculated using chi-square tests or Fisher exact tests where appropriate.

a Adjusted estimates are from an exploratory reduced logistic regression model including sex, age group, chronic medical condition status, seasonal influenza vaccination, and pre-ritual ARI status. Variables not included in the reduced adjusted model are presented with unadjusted estimates only. Pre-ritual ARI was defined using the final study case definition of 234/541 symptomatic participants. The reduced model showed limited explanatory power; adjusted estimates should therefore be interpreted cautiously. Abbreviations: ARI, acute respiratory illness; CI, confidence interval; df, degrees of freedom; OR, odds ratio.

Symptom occurrence did not differ significantly by sex. Symptoms were reported by 93/179 male pilgrims (52.0%) and 164/362 female pilgrims (45.3%, χ²(1) = 2.12, *P* = 0.145). Symptom occurrence also did not differ significantly by age group (χ²(3) = 2.29, *P* = 0.515), ranging from 43.4% among pilgrims aged 40-49 years (59/136) to 52.7% among those aged ≥60 years (59/112).

Post-return ARI was more frequent among pilgrims who had reported ARI during the pre-ritual period in Saudi Arabia. Among those with pre-ritual ARI, 123/234 (52.6%) reported ARI within 14 days after return, compared with 134/307 (43.6%) among those without pre-ritual ARI (χ²(1) = 4.23, *P* = 0.040; Fisher exact *P* = 0.046).

#### Bivariate associations

Unadjusted associations between participant characteristics and self-reported ARI within 14 days after return to South Africa are shown in Table 3.

Pre-ritual ARI was significantly associated with post-return ARI in unadjusted analysis. Pilgrims who reported ARI during the pre-ritual period in Saudi Arabia had higher odds of reporting ARI within 14 days after return than those without pre-ritual ARI (OR 1.43; 95% CI 1.02-2.01; *P* = 0.040).

No statistically significant associations were observed between post-return ARI and sex, age group, marital status, education level, smoking history, baseline self-rated health, chronic medical condition status, walking distance capacity, or seasonal influenza vaccination. Seasonal influenza vaccination was not significantly associated with post-return ARI in unadjusted analysis (OR 1.28; 95% CI 0.90-1.82; *P* = 0.155).

Weight status showed a borderline, non-significant association with post-return ARI (χ²(2) = 5.89, *P* = 0.053). Pilgrims with obesity reported a higher proportion of post-return symptoms than pilgrims with normal weight or underweight (10/14; 71.4% vs 151/341; 44.3%), but this estimate was imprecise because of the small number of participants with obesity.

#### Exploratory multivariable analysis

Given sparse data in several exposure categories and unstable estimates in the fully adjusted model, the post-return multivariable analysis was restricted to a reduced set of *a priori* variables: sex, age group, chronic medical condition status, seasonal influenza vaccination, and pre-ritual ARI status.

In this exploratory adjusted model, pre-ritual ARI remained associated with post-return ARI. Pilgrims who reported ARI during the pre-ritual period in Saudi Arabia had higher adjusted odds of reporting ARI within 14 days after return to South Africa than those without pre-ritual ARI (aOR 1.46; 95% CI 1.03-2.07; *P* = 0.033).

No adjusted associations were observed for sex, age group, chronic medical condition status, or seasonal influenza vaccination. Seasonal influenza vaccination was not associated with post-return ARI after adjustment (aOR 1.20; 95% CI = 0.85-1.71; *P* = 0.293).

The reduced model explained a small proportion of outcome variation, and the overall likelihood-ratio test was not statistically significant (likelihood-ratio χ²(7) = 10.50; *P* = 0.162; pseudo *R*² = 0.014). Adjusted estimates should therefore be interpreted cautiously.

## Discussion

This retrospective observational cohort study found a high burden of self-reported modified syndromic ARI among South African pilgrims across two phases of the 2025 Hajj travel continuum. During the pre-ritual period in Saudi Arabia, 234/541 (43.3%) pilgrims reported two or more self-reported symptoms meeting the modified syndromic ARI case definition. Within 14 days after return to South Africa, 257/541 (47.5%) pilgrims reported modified syndromic ARI. These findings indicate that respiratory symptoms were common both before the core 5-day Hajj rites and after return, but the pattern of associated factors differed between the two periods. Older age was associated with lower adjusted odds of pre-ritual ARI, whereas seasonal influenza vaccination was associated with higher adjusted odds of pre-ritual symptom-defined ARI; pre-ritual ARI was also modestly associated with post-return ARI in the exploratory adjusted model. Because the outcome was syndromic and self-reported, these findings should be interpreted as patterns of respiratory symptom occurrence rather than laboratory-confirmed respiratory infection.

The findings should be interpreted in relation to the modified syndromic ARI definition. The study did not use a single-symptom threshold; ARI required two or more self-reported symptoms during the relevant recall period. This approach improved specificity compared with classifying cough, rhinorrhea, or nasal congestion alone as ARI. However, the definition remained syndromic and retrospective. It did not require measured fever, exact symptom onset date, or laboratory confirmation and should therefore not be interpreted as ILI, COVID-19, or pathogen-confirmed respiratory infection. This distinction is important when interpreting the counterintuitive associations observed for older age and influenza vaccination.

The respiratory symptom burden observed in this cohort is consistent with previous South African Hajj studies, although differences in timing, case definition, and outcome measurement affect direct comparability. In a 2023 retrospective cohort study of South African pilgrims, 577 of approximately 3500 accredited pilgrims responded; 259/577 (45%) reported illness during their stay in Saudi Arabia, and 293/577 (51%) reported illness within 7 days of returning to South Africa [10]. Among those reporting illness in that study, respiratory-linked symptoms accounted for 95% of illness during the Saudi Arabia phase and 99% after return [10]. The current study’s pre-ritual ARI occurrence of 43.3% is therefore close to the 2023 estimate during the Saudi Arabia phase, while the post-return ARI occurrence of 47.5% is slightly lower than the 2023 post-return estimate. This similarity is plausible given the shared South African pilgrim context, but the present study used a more specific syndromic ARI outcome and separated the pre-ritual period from post-return illness, whereas the 2023 study reported broader morbidity categories.

The current findings also align with the 2017 South African Hajj cohort, although the magnitude differs. That longitudinal survey included 1138 adult South African pilgrims and collected data during and after Hajj [9]. Approximately 65% of respondents reported illness during Hajj, and 40% reported illness after return to South Africa; among those who were ill, respiratory symptoms were reported by 70.2% during Hajj and 82.2% after return [9]. Compared with that cohort, the present study found lower pre-ritual ARI rates than the 2017 overall illness estimate during Hajj, but a higher post-return respiratory symptom rate than the 2017 post-Hajj overall illness estimate. These differences are likely explained by the different denominators and definitions used: the 2017 study reported overall illness and respiratory symptoms among ill pilgrims, while the current study estimated syndromic ARI directly among all respondents. The comparison indicates that respiratory morbidity remains a recurrent feature of South African Hajj participation, but measured burden depends strongly on the recall period, phase of pilgrimage assessed, and whether respiratory symptoms are measured as a primary outcome or as a subset of general illness.

International studies similarly show that respiratory symptoms are common among Hajj pilgrims, but reported estimates vary widely by population and measurement approach. In a Malaysian cross-sectional survey of 387 returning Hajj pilgrims, cough was reported by 91.5%, runny nose by 79.3%, fever by 59.2%, and sore throat by 57.1%; only 3.6% reported none of these symptoms [16]. A French cohort of 485 pilgrims followed between 2014 and 2017 found that more than 80% experienced at least one respiratory symptom during Hajj, while ILI was reported by 16.9% [17]. Another French surveillance study of 382 pilgrims during 2012-2014 reported cough in 80.9% of participants, with frequent accompanying sore throat, rhinitis, hoarseness, myalgia, and subjective fever [18]. In an international study during the 2022 Hajj season, among 2913 Hajj pilgrims, cough was reported by 1173 (40.4%) participants. Chronic diseases were more common among those with cough (53.3%), and the most frequent conditions were diabetes and hypertension. Among pilgrims with cough, sore throat was the most common associated symptom (30.8%), while 10.3% reported no additional symptoms. Age, nationality, and chronic disease were significant predictors of cough [19]. Estimates from the international Hajj study during the 2022 Hajj season are higher than the 43.3% pre-ritual ARI occurrence observed in the current study. The difference is likely due to the current study’s phase-specific measurement before the core 5-day rites, whereas several comparator studies assessed symptoms across the full pilgrimage or immediately before return. Variation may also reflect differences in country of origin, accommodation density, duration of stay, exposure to crowds, climate adaptation, symptom ascertainment, and case definition.

The lower adjusted odds of pre-ritual ARI among pilgrims aged ≥60 years are noteworthy and should not be interpreted as biological protection from respiratory infection. The association persisted after adjustment for sex, chronic medical condition status, walking distance capacity, and influenza vaccination. Additional exploratory analyses showed no significant age-group differences in walking distance capacity or physical training before Hajj, while older pilgrims had a higher prevalence of chronic medical conditions and higher influenza vaccination uptake. This suggests that the lower odds among older pilgrims were not explained by measured functional capacity, absence of chronic disease, or lower vaccination uptake. A more plausible explanation is differential exposure and reporting: older pilgrims may have paced activities more conservatively, avoided non-essential movement or informal crowd exposure, and differed in symptom perception or recall. The finding should therefore be interpreted as a phase-specific pattern of reported syndromic ARI rather than evidence of lower biological susceptibility.

The association between seasonal influenza vaccination and higher adjusted odds of pre-ritual symptom-defined ARI requires careful interpretation. South Africa uses the Southern Hemisphere seasonal influenza vaccine formulation, and vaccination is generally offered before the winter influenza season. In 2025, South Africa’s influenza season began in late March, overlapping with the pre-Hajj preparation period [12,13]. The observed association should not be interpreted as evidence of vaccine failure or harm, as the outcome was modified syndromic ARI rather than laboratory-confirmed influenza. In the Malaysian study of 387 returning pilgrims, 72.0% reported influenza vaccination before departure, but influenza vaccination was not associated with respiratory symptoms [16]. In the French 2014-2017 cohort, multiple respiratory pathogens were detected among pilgrims, with acquisition of rhinovirus, seasonal coronaviruses, influenza viruses, *H. influenzae, S. pneumoniae, Klebsiella pneumoniae*, and *Staphylococcus aureus* documented after Hajj [17]. Similarly, molecular surveillance among 129 French pilgrims during the 2013 Hajj showed an increase in respiratory virus detection from 21.5% before Hajj to 38.8% after Hajj, with acquisition of rhinovirus, coronavirus 229E, and influenza A(H3N2) during the stay in Saudi Arabia [6]. These data demonstrate that syndromic respiratory illness during Hajj reflects multiple viral and bacterial exposures, most of which would not be prevented by influenza vaccination. The higher odds among vaccinated pilgrims in the current study may therefore reflect residual confounding, health awareness, indication for vaccination, differential symptom recognition, or reporting behavior rather than a biological effect of vaccination on ARI risk.

The post-return phase showed a different epidemiological pattern. Post-return ARI affected 47.5% of respondents and was modestly associated with pre-ritual ARI in both unadjusted and exploratory adjusted analyses. Pilgrims with pre-ritual ARI had higher adjusted odds of reporting ARI within 14 days after return than those without pre-ritual ARI, but the effect size was modest, and the reduced model explained little outcome variation. This suggests phase overlap rather than a clearly separable post-return illness process. The association may reflect the persistence of symptoms acquired before or during the pilgrimage, new exposures during the core rites or during return travel, individual susceptibility to recurrent respiratory symptoms, or differential recall among participants who were symptomatic in more than one period. This interpretation is supported by molecular surveillance among French pilgrims, where many participants with post-Hajj respiratory pathogens also reported symptoms during their stay in Saudi Arabia, and a substantial proportion remained symptomatic at the time of post-Hajj sampling [6]. The present study could not distinguish persistent symptoms from new episodes; therefore, the post-return association should be interpreted as continuity or overlap in symptom reporting rather than as confirmed onward infection.

The absence of adjusted associations between post-return ARI and sex, age, chronic medical condition status, or seasonal influenza vaccination also requires balanced interpretation. In the 2023 South African Hajj cohort, chronic conditions were associated with higher adjusted odds of post-return illness [10]. In contrast, chronic-condition status was not associated with post-return ARI in the current reduced exploratory model. This difference may be explained by differences in outcome definition, timing, model specification, and measured covariates. The 2023 study assessed overall post-Hajj illness within 7 days of return, whereas the present study examined syndromic ARI within 14 days of return. Similarly, the lack of association between influenza vaccination and post-return ARI in the current study is consistent with the broad syndromic outcome and pathogen diversity documented in Hajj cohorts [5,6,17]. The borderline unadjusted association between obesity and post-return ARI should also be interpreted cautiously because only 14 respondents were classified as having obesity, producing imprecise estimates and limiting meaningful inference.

When analyzed collectively, the findings suggest that respiratory symptoms among South African Hajj pilgrims are substantial but not uniform across the travel continuum. The pre-ritual phase appeared to show more distinct associations, particularly with age and influenza vaccination status, while the post-return phase was characterized by modest overlap with pre-ritual ARI and limited explanatory power in the adjusted model. This pattern is consistent with the broader Hajj literature, which shows high but variable respiratory symptom estimates across pilgrim populations and repeated detection of multiple respiratory pathogens after Hajj [5,6,17]. For the current study, the main interpretive contribution is therefore not simply that ARI was common, but that the timing of symptom assessment materially influenced which associations were observed. Distinguishing pre-ritual and post-return symptom periods provides a more nuanced account of Hajj-associated respiratory morbidity than a single aggregate post-Hajj measure.

### Strengths and limitations

This study has several strengths. First, it provides population-specific evidence on self-reported ARI among South African pilgrims participating in the 1446H/2025 Hajj, a population that remains underrepresented in the Hajj respiratory illness literature. Second, the study assessed ARI across two clearly defined phases: the pre-ritual period in Saudi Arabia before commencement of the core 5-day Hajj rites, and the first 14 days after return to South Africa. This phase-specific approach allowed respiratory symptoms occurring before the core rites to be distinguished from those reported after return, rather than treating Hajj-associated illness as a single aggregate outcome.

Third, recruitment was open to all accredited South African pilgrims through SAHUC communication channels, reducing the clinic-based or hospital-based ascertainment bias that may occur when only pilgrims seeking healthcare are included. The final respondent cohort represented 21.6% of the eligible accredited population, which was adequate for estimating overall symptom occurrence and examining the main pre-ritual associations. Fourth, the analysis used a consistent modified syndromic ARI case definition across both outcome periods and explicitly separated pre-ritual and post-return analyses. The statistical approach also addressed collinearity among functional-capacity variables and treated the post-return adjusted model as exploratory because of sparse data and limited explanatory power.

Some limitations should be considered when interpreting the findings. Respiratory illness was defined using self-reported symptoms without laboratory confirmation. The outcomes therefore represent modified symptom-defined ARI rather than pathogen-confirmed respiratory infection, limiting pathogen-specific attribution and preventing conclusions about influenza vaccine effectiveness. The modified syndromic ARI definition improved specificity compared with a single-symptom definition by requiring two or more symptoms. However, diagnostic specificity remained limited because symptoms were self-reported retrospectively and were not laboratory-confirmed. Fever was subjective and not objectively measured. Non-infectious causes of symptoms, including allergic rhinitis, asthma, chronic respiratory disease, environmental irritation, heat exposure, dehydration, or fatigue, could not be excluded. The absence of measured temperature and exact symptom onset date also meant that standard ILI or severe acute respiratory infection surveillance definitions could not be applied.

Symptom data were collected retrospectively approximately 1 month after Hajj. Recall bias is therefore possible, particularly in distinguishing symptoms occurring during the pre-ritual period from those occurring after return. The study also could not determine exact symptom onset, duration, severity, healthcare utilization, or whether post-return symptoms represented persistent illness, a new episode, or repeated exposure. This is important because pre-ritual ARI was associated with post-return ARI, but the study design does not allow this association to be interpreted as confirmed persistence or onward infection. Although the lower odds of pre-ritual ARI among pilgrims aged ≥60 years persisted after adjustment for measured functional capacity, chronic medical condition status, sex, and influenza vaccination, the study did not measure contact intensity, daily mobility, accommodation density, caregiving roles, crowd exposure, or symptom attribution by age group. The mechanism underlying the age association therefore remains uncertain.

Influenza vaccination status was self-reported and may have been subject to recall or misclassification. The study could not reliably distinguish Hajj-specific vaccination from routine seasonal vaccination or vaccination received because of underlying risk status. This limits interpretation of the observed association between influenza vaccination and pre-ritual modified syndromic ARI. Chronic medical condition status was also captured as a self-reported binary variable rather than as individual diagnoses, and the study was not powered to examine disease-specific effects. Obesity was analyzed separately as weight status and was not included in the chronic-condition composite. In addition, COVID-19 vaccination data were excluded due to substantial missingness and inconsistent reporting, and key behavioral and exposure variables, such as mask use, hand hygiene, crowding in accommodation, transport exposures, contact intensity, and prior Hajj experience, were unavailable. Residual confounding is therefore likely.

The voluntary response proportion of 21.6% introduces potential non-response bias. Individual-level demographic, clinical, or vaccination data were not available for non-respondents, so respondents could not be formally compared with the full accredited pilgrim population. Participation also required access to an electronic device, which may have underrepresented digitally excluded pilgrims. Finally, although the sample was sufficient for estimating overall ARI occurrence, the post-return multivariable model had limited explanatory power. Adjusted post-return estimates should therefore be interpreted cautiously and regarded as exploratory rather than definitive.

## Conclusion

Modified syndromic ARI was common among South African pilgrims during the pre-ritual period in Saudi Arabia and within 14 days after return to South Africa. Pre-ritual ARI was reported by 234/541 pilgrims, while post-return ARI was reported by 257/541 pilgrims, indicating a substantial symptom burden across both phases of the Hajj travel continuum. The pattern of associated factors differed by phase: older age was associated with lower adjusted odds of pre-ritual ARI, seasonal influenza vaccination was associated with higher odds of pre-ritual modified syndromic ARI, and pre-ritual ARI remained modestly associated with post-return ARI in exploratory adjusted analysis.

These findings should be interpreted in the context of a retrospective self-reported modified syndromic outcome and should not be used to infer pathogen-specific infection, ILI, COVID-19, influenza vaccine effectiveness, or vaccine failure. Rather, they suggest that respiratory symptoms among South African pilgrims are common, phase-specific, and only partly explained by baseline demographic, clinical, behavioral, and vaccination-related factors. The association between pre-ritual and post-return ARI may reflect overlapping or persistent symptoms, repeated exposure, individual susceptibility, or reporting patterns rather than a clearly defined new infection episode.

Future Hajj respiratory illness studies should incorporate repeated symptom assessment with laboratory confirmation to distinguish syndromic illness from pathogen-specific infection and to clarify the timing of symptom onset, persistence, and recovery. Larger multicountry cohorts with data on accommodation, crowd exposure, transport, mask use, hand hygiene, vaccination timing, comorbidity, and healthcare utilization would strengthen the interpretation of phase-specific respiratory illness patterns and allow more robust comparison across pilgrim populations.

## Funding

This research did not receive any specific grant from funding agencies in the public, commercial, or not-for-profit sectors.

## Ethics approval and consent to participate

This study was reviewed and approved by the Human and Social Sciences Research Ethics Committee of the University of KwaZulu-Natal, South Africa (HSSREC/00008459/2025). Permission to access the accredited South African Hajj pilgrim cohort was granted by the SAHUC. Participation was voluntary, and all participants provided digital informed consent before completing the questionnaire. The study was conducted in accordance with the principles of the Declaration of Helsinki.

## Informed consent statement

Digital informed consent was obtained from all participants before completion of the electronic questionnaire.

## Consent for publication

Not applicable. This manuscript does not contain identifiable personal details, images, videos, or individual-level clinical information.

## Availability of data and materials

The dataset generated and analyzed during the current study is not publicly available because it contains participant-level health and travel information. De-identified data may be made available from the corresponding author on reasonable request, subject to ethics approval conditions and appropriate data-sharing agreements.

## Declaration of generative AI and AI-assisted technologies in the writing process

During the preparation of this manuscript, generative AI was used to assist with language editing, structural refinement, and clarity of expression. The authors reviewed and edited all AI-assisted content and take full responsibility for the accuracy, integrity, and final content of the manuscript.

## CRediT authorship contribution statement

**Ozayr Mahomed:** Writing – review & editing, Writing – original draft, Formal analysis, Conceptualization. **Salim Parker:** Writing – review & editing, Methodology, Conceptualization. **Jaffar A. Al-Tawfiq:** Writing – review & editing.

## Declaration of competing interest

The authors have no competing interests to declare.
